# Effects of Feeding Frequency for Nile Tilapia Fingerlings (*Oreochromis niloticus*)

**DOI:** 10.1155/2022/1053556

**Published:** 2022-11-22

**Authors:** Aldo Felipe Fava, Gildete de Souza Bezerra, Dacley Hertes Neu, Fabio Bittencourt, Altevir Signor, Kerolay Valadão Carvalho, Ricacio Luan Marques Gomes, Wilson Rogério Boscolo

**Affiliations:** ^1^Universidade Estadual do Oeste do Paraná, Centro de Engenharias e Ciências Exatas, Rua da Faculdade, 354, Toledo, PR, Brazil; ^2^Universidade Federal da Grande Dourados, Faculdade de Ciências Agrárias, Rodovia Dourados-Itahum, Km 12, Cidade Universitária, Dourados, MS, Brazil; ^3^Centro de Aquicultura da Universidade Estadual Paulista Julio de Mesquita Filho, Jaboticaba, SP, Brazil

## Abstract

This study is aimed at assessing the feeding frequency for tilapia fingerlings. 240 fishes were randomly distributed in 24 containers. The feeding consisted of six frequencies: 4 (F4), 5 (F5), 6 (F6), 7 (F7), 8 (F8), and 9 (F9) times a day. The weight gain was significantly higher in F5 and F6 when compared to F4 (*p* = 0.0409 and 0.0306, respectively). Feed intake and apparent feed conversion did not differ between treatments (*p* = 0.129 and *p* = 0.451). About water quality, the nitrogen levels in the water from the treatments differed between F4 and F5 (*p* = 0.0478) and F4 and F6 (*p* = 0.0283) and for P between F4 and F6 (*p* = 0.0215) and F4 and F9 (*p* = 0.0432). The *x*^2^ test showed a dependence relationship between feed frequencies and the muscle fibers frequency (*p* < 22 × 10^−17^), prevailing fibers between 10-20 micrometers in F4, F5, F6, and F7 and 30-40 in F8 and F9. The area of the hepatocytes differed only between F5 and F9, while the nucleus area did not. Partial net revenue differed in 10% between F5 and F4 (*p* = 0.0812) and between F6 and F4 (*p* = 0.0568). In conclusion, fingerlings fed 5 to 6 times a day have better zootechnical and partial recipes.

## 1. Introduction

The demand for food, especially that of protein origin, has grown as a result of the increase in the world population, and the aquaculture has met part of this demand due to its expressive growth, showing potential to overcome this problem, providing high-value proteins [1]. In this context, *O. niloticus* is the third most produced fish species in the world corresponding to 8.3% of production [2] and the most produced in Brazil (57%) [3], which occurs because the specie meets the demand for greater production in small spaces, this reduces the production cycle, and it is rustic and easy to reproduce, making it widely studied, with the main focus on improving the efficiency of fish farms [4, 5].

Therefore, feeding is decisive for the viability of these systems, as it represents between 40 and 60% of the total costs [6] and can reach 70% [7]. It is considered that the influence of diet on growth is not limited to the choice of ingredients that will compose it, the frequency with which it is offered also alters the utilization rate of nutrients [8].

In this way, feeding frequencies vary between the species, production systems, and stage of development in which the fish are [9]. Fish must have access to food in order to be satiated; however, if there is overfeeding, the excess raises costs and worsens feed conversion ratio and there will be an increase in costs [10]. As for the species, in omnivorous with small stomachs, such as *O. niloticus*, the search for food occurs more frequently due to their limited storage capacity [11], not being able to consume enough in smaller frequencies like other carnivorous species or larger stomach species. As for postlarvae and fingerlings, as they have a more active metabolism, their digestive tract is not fully developed, and they seek food more often than juveniles [12], so feeding at the ideal frequency favors growth and optimizes the use of the feed. According to study of Fattah et al. [13], increasing feeding frequency for tilapia may increase the chances of ingestion and feeding behavior, improving swimming behavior, and can reduce aggressive behavior. However, the ideal feeding frequency for tilapia fingerlings has not been established.

Regarding the organism of these fingerlings, more specifically the liver, where the processing of food occurs, it has a limited capacity for this, and its tissue can be compromised when its capacity is exceeded. This fact explains why overfeeding (high frequencies) can reduce feed utilization, worsening feed conversion ratio, leading to accumulation of fat and waste of nutrients (rich in phosphorus and nitrogen), impairing economic performance, and increasing environmental impact [8, 9].

Therefore, it is necessary to investigate the feeding frequencies with the aim of evaluating their effects on fingerlings, as this parameter impacts the intake and growth, feed conversion ratio, and water quality [8, 12].

## 2. Material and Methods

The experiment was carried out at the Gemaq, Aquaculture Management Study Group, Aquaculture Laboratory at Unioeste - State University of Western Paraná (Toledo), before being submitted to the Ethics Committee on Animal Use (Protocol number 01-19) and approved, in accordance with the technical norms of biosafety and ethics for the use of animals in experiments.

For the experiment execution, 24 polyethylene rectangular boxes (with sides of 89 cm and 56 cm and depth of 48.5 cm) with a capacity of 180 liters were used with a constant aeration system. The experimental design was completely randomized, evaluating six feeding frequencies (4, 5, 6, 7, 8, and 9 times a day) with four replicates; the experimental unit was composed of a box containing ten fingerlings. Five hundred fingerlings of the same spawn were acquired from a commercial fish farm called Dal Bosco Fish Farm, in the region of west Paraná, in the municipality of Toledo (near of the site of the present study) and transported to the laboratory in isothermal transport boxes with constant oxygenation. Afterwards, their mean weight (0.793 g) was determined, distributing the fishes in 24 boxes and keeping the mean weight of them in 0.793 ± 0.05 g. The duration of the feeding trial was 30 days.

### 2.1. Feed Making

The rations were formulated (Table 1) in order to meet the nutritional demand of the species at the stage of development used, according to the Brazilian Tilapia Nutrition Tables [14]. Each ingredient in the diet was balanced using the SuperCrac® 5.7 Master program [15]. To prepare the diet, the ingredients were ground in a hammer mill (Vieira®, MCS 280, Tatui, SP), with a 0.3 mm sieve. For extrusion, an Exteec brand extruder, EX 500, Ribeirão Preto, SP, in a 1 mm die was used. After this process, the bromatological analysis of the ration was performed (Table 1), in which the dry matter, crude protein, ether extract, mineral matter, and gross energy content were evaluated, according to the methodology described by AOAC and adapted by the Adolfo Lutz Institute [16].

### 2.2. Handling during the Experiment

The feeding management of the fingerlings consisted of offering food 4, 5, 6, 7, 8, and 9 times a day, until apparent satiety, from 8 : 00 AM to 6 : 00 PM. The option for *ad libitum* feeding was made to verify the consumption capacity of the fingerlings as a function of the frequencies and for to know which one allows the maximum performance, what would not be possible with feeding by percentage of live weight. All the ration consumed was weighted to calculate the feed intake (FI) and after the apparent feed conversion (AFC), that is, the relationships between the FI and weight gain (WG).

The *ad libitum* feed was offered as follows: feed was offered until the fish stopped feeding; after reaching the last box to be fed on time, the first box was observed; and if there was no more interest from the animals, it was stopped the feeding. Otherwise, they were fed again, until satiety. And it was made in all the box. The cleaning of the experimental units was carried out by siphoning the bottom of the polyethylene boxes, with the removal of freshwater, 30% of the volume; in the afternoon, after the last feeding, and in sequence, the volume of the box has been completed with water of similar temperature. There was no water recirculation during the experiment; the water in each experimental unit was replenished individually, to guarantee the independence of the experimental units regarding the water quality parameters.

Weekly, water quality parameters such as pH, dissolved oxygen (mg.l^−1^), and electrical conductivity (*μ*S.cm^−1^) were checked, as well as daily temperature (°C) in the morning and afternoon periods. At the beginning and end of the study, water was collected from the experimental units and stored in dark polyethylene bottles and cooled for later analysis of total nitrogen (TN) and total phosphorus (TP) according to the AOAC methodology adapted by the Adolfo Lutz Institute [16].

At the end of the experimental period, the specimen remained fasted for 12 hours and were anesthetized with eugenol (100 ml/l of alcohol) and weighed and measured to determine the zootechnical parameters. It was evaluated the final biomass (g), total length (TL), standard length (SL), weight gain (WG = [final weight (g)–initial weight (g)]), and apparent feed conversion (AFC = [food consumed (g)/weight gain (g)]) of the animals submitted to the different treatments.

### 2.3. Histology

The fish, randomly selected for organ collection, were euthanized in eugenol solution, and the livers of three animals per experimental unit were collected for the hepatosomatic relation (HSR%), totaling 12 fish per treatment. The HSR% was calculated by the ratio of liver weight (g) to fish body weight (g), multiplied by 100. The livers of two fish were removed and washed in physiological solution (NaCl 0.9%), and samples were taken from the median portion of the left lobe. The liver samples were fixed in Alfac solution [17] and 12 hours later transferred to flasks containing 70° alcohol, and then, the liver samples were dehydrated, diaphonized, and embedded in paraffin, to obtain 6 *μ*m thick rotating microtome (MICROM, International Gmbh 69190, Walldorf, Germany) sections, and two slides with three histological sections per liver were mounted.

The liver samples were processed and stained by the method of hematoxylin and eosin (HE), for general morphological analysis. Morphometric analyses of the liver were performed from images captured in a P1 optical microscope Olympus BX 50 (Manila, Philippines) coupled with a camera (Olympus PMC 35 B, Berlin, Germany), using a 40x objective. The calculations performed were the area of the hepatocyte and its nucleus (area = *π*∗radius^2^), the radius being obtained by the diameter measured using the ImageJ software (version 4.5, Media Cybernetics, USA).

### 2.4. Liver Integrity

The analyses of morphological changes were qualitatively assessed using the injury index according to Bernet et al. [18], calculated from the index Bernet = *Σ* importance factor (*w*) × score (*α*); therefore, these alterations were classified into three importance factors (*w*), (1) reasonable injury, (2) moderate injury, and (3) irreversible damage, leading to partial or total organ loss. Each histopathological alteration through scores (*α*) that varied from 0 to 6 depends on the degree of alteration, being (0) without alteration, (2) little occurrence, (4) moderate occurrence, and (6) serious injury. To determine the lesions, a table was developed for the respective study, indicating the main histopathological lesions found.

### 2.5. Muscle Histology

Muscle samples were collected from the dorsal white muscle, above the lateral line, of two fish from each experimental unit for histology. Afterwards, they were fixed in 10% buffered formaldehyde; their cuts were made in a rotating microtome with a thickness of 6 *μ*m. Thus, two slides with three histological sections per sample were prepared. The hematoxylin-eosin (HE) technique was used to color the slides in order to measure the diameter of muscle fibers. From the HE-stained slides, the smallest diameter of each fiber was measured, with 200 fibers per slide, as described in a study by Assis et al. [19]. All histological analyses were performed with the aid of ImageJ image analysis software (https://imagej.net/Fiji) [20]. The frequency and distribution of muscle fibers were classified into four diameter classes (*D*) (0-10 *μ*m, 10-20 *μ*m, 20-30 *μ*m, and 30-40 *μ*m).

### 2.6. Partial Net Revenue

The partial economic analysis was carried out determining the production costs, considering only the period of the studied phase (fry to juveniles). The costs considered in the partial economic evaluation were the purchase of fingerlings and feed. The profit percentage was based only on the sale price of the fish, and then, the gross revenue was calculated. To calculate the partial net revenue (PNR), the equation used was PNR = GR − (Fg + Fd), where GR is the gross revenue corresponding to the sale price of the fish according to its final weight, Fd is the total cost of feeding, and Fg is the purchase cost of fingerlings.

### 2.7. Statistical Analysis

Initially, it was verified whether the variance of the data reached the presuppositions for carrying out the univariate analysis of variance (ANOVA). The normality assumption was verified by the Shapiro-Wilk test and the homogeneity by the Levene test, and these assumptions were reached when the *p* value was above 5% (*p* > 0.05).

When the assumptions were reached, the data were submitted to ANOVA, considering that there was a difference when *p* value was below 5% (*p* < 0.05), and in this case, they were submitted to the Tukey test for the comparison of means; this also using the 5% level. All statistical analysis was performed using the statistical program R [21].

To assess the influence of feeding frequencies on the frequency of occurrence of muscle fibers in different diameters, a chi-square distribution was performed. This methodology was chosen because it deals with two qualitative variables, and in this case, the use of ANOVA is not justified. Subsequently, correspondence analysis was carried out, ordering the rows and columns of the two variables simultaneously and explaining the relationship between the factors [22].

Several biological and chemical variables were measured during the study to evaluate the feeding frequency, and for this reason, a principal component analysis (PCA) was carried out in order to analyze the various variables in a more understandable way, since the PCA is multivariate and performs an exploration of the correlation structure between the variables, producing a group of components that reflect the original set [23]. With this, it was possible to reduce the size of the data and to reject the components with the less variation. It was performed using R software [21] with multcomp packages [24].

## 3. Results and Discussion

During the experimental period (30 days), no significant differences were observed between the treatment means regarding the water quality control variables (Table 2): temperature (°C), dissolved oxygen (mg/l), electrical conductivity (*μ*S/cm^2^), and pH.

Variables of temperature (°C), dissolved oxygen (mg/l), electrical conductivity (*μ*S/cm^2^), and pH were in the ideal conditions for the development of the species at this stage [25]. Factors such as feeding frequency can configure a direct correlation with the water conditions of the growing environment through the efficiency of food utilization and the amount consumed. The interferences that occurred in the performance were not enough to change these parameters, so that all treatments produced conditions that allow the good development of the tilapia, implying that the possible changes in performance occurred due to the use of the food.

The TN contents differed statistically by ANOVA (*p* = 0.0273), and by Tukey's test, it was possible to observe a statistical difference between F4 and F5 (*p* = 0.0478) and between F4 and F6 (*p* = 0.0283). There was also a statistical difference in PT values (*p* = 0.014), for Tukey F4 and F6 and F4 and F9 differed (*p* = 0.0215 and *p* = 0.0432, respectively), with F5 and F4 showing statistical difference only at the level 10% (*p* = 0.0579) (Table 3).

The TP contents were lower in F4 because the maximum consumption capacity in this treatment was not reached, and with the lower feed intake, the amount of manure and remaining feed was lower. Regarding TN, although smaller in F4, it did not differ statistically from F7 and F8 but differed from F9. In F9, the higher frequency meant that the animals received the feed when they were already satiated, reducing the use. In F7 and F8, this occurred, but with less impact, as the frequency was lower. These nutrients are of great importance in relation to the effluent, as they are the main limiting factors for the occurrence of eutrophication in water bodies [26].

Regarding weight gain, there was a statistical difference between F4 and F5 (0.0409) and F4 and F6 (0.0306) and at the 10% level between F4 and F9 (*p* = 0.0995). FI did not have the same behavior, as well as AFC (*p* = 0.451). TL differed between F4 and F5 (*p* = 0.0149) and between F4 and F6 (*p* = 0.0303) and SL between F4 and F5, F4 and F6, and F4 and F9 (*p* = 0.0089, *p* = 0.0162, and *p* = 0.0257, respectively). For HSR, there was no statistical difference between treatments (*p* = 0.948).

Fish fed 4 times a day gained less weight than higher frequencies. No differences in WG from 5 to 9 times a day were observed. In a study by Tian et al. [27], the authors observed feeding frequencies of 1, 3, and 6 times a day and noted that weight gain increased from 1 to 3 times a day and declined between 3 and 6. Although the species and stage of development are different and, consequently, the frequencies are different, it is observed that the gain no longer occurs after a certain frequency, which occurs in the present study (Table 4), with the greatest weight gain (WG) and final biomass (FB) occurring when tilapia were fed between five and six times a day when compared to four times, with the gain not significantly different between the higher frequencies (seven, eight, and nine times). Booth et al. [28] observed that short intervals between feedings make the food pass faster through the digestive tract at higher frequencies, which reduces the use of food, corroborating the fact that, in the present study, the higher frequencies did not cause higher WG.

Although the FI did not differ statistically in the experimental conditions, it is possible to see that treatments F5 and F6 had the highest consumption, a result that can be corroborated by their greater weight gain. Treatments F7, F8, and F9 did not maintain the growth trend (which occurred from F4 to F5) with higher frequencies. Motor and enzymatic activity increases in periods close to fish feeding [29], favoring absorption at certain times; however, in F7, F8, and F9, the interval for the occurrence of this phenomenon was shorter and may have limited use of the feed, due to the shorter preparation time of the organism between one feeding and another. Therefore, the highest frequency maintained the growth between 5 and 9 times a day, but considering the smaller workforce, 5 times becomes economically favorable. According to Tian et al. [27], low feeding frequencies reduce growth, but high frequencies burden and reduce the use of feed.

The treatments did not differ statistically from each other regarding apparent feed conversion (Table 4). This fact occurred due to the higher consumption in treatments F5 and F6 that had the highest WG; as AFC is the relationship between FI and WG, it remained constant, with no statistical difference, through feed conversion, in the use of rations. However, observing only the FI, it is observed that F4 had a reduced consumption in relation to F5, F6, and F9, being, in this case, a result of the higher frequency of offers in such treatments, although the sample size does not allow such inference.

Another aspect that may be related to increased growth in F5 and F6 is enzymatic activity. In a study by Silva et al. [8], it was found that enzymatic activity is related to feeding efficiency, and these authors tested feeding frequencies of one, three, five, and seven times a day and recorded maximum activity of proteolytic, amylolytic, and lipolytic digestive enzymes of three to five times a day, which, in the present study, may explain the greater gains in frequencies between five and six. Although this activity has not been reflected in AFC, it is probably due to the period (only 30 days) for the tilapia to reach the juvenile stage (10 g), which was longer in the study mentioned (60 days). The difference in consumption between the frequencies also explains the WG, as in a study by Santos et al. [30], the authors observed that, with the same amount of feed offered in the treatments (in other words, offer by live weight), tilapia fingerlings did not differ statistically in WG between 4 and 6 times a day. In the present study, feeding the will, feed intake increases at frequencies greater than 4, although the sample size did not allow for the statistical response, which should be detailed in future studies.

There was no difference in the HSR, which shows that, although the efficiency of nutrient absorption was different between treatments, it did not change in the HSR, which is of great importance for the nutritional status of fish, taking into account the importance of the liver for the digestion and processing of nutrients [31]. Therefore, until the assessed phase, liver health was not impaired by the frequency of feeding.

The *x*^2^ test showed that there is a dependency relationship between the food frequencies and the frequency of occurrence of the muscle fibers (*p* < 22 × 10^−17^). This analysis was made with the frequencies observed in Table 5.

**Figure 1:**
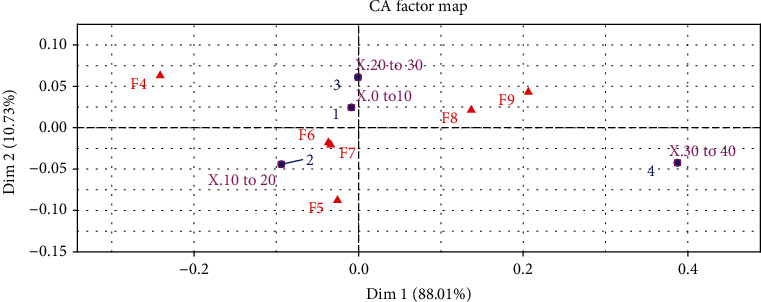
Graph showing the association between the frequency of feeding (F4, F5, F6, F7, F8, and F9) and the occurrence of muscle fibers in different classes (0-10, 10-20, 20-30, and 30-40 *μ*m). F4: 4 times daily; F5: 5 times daily; F6: 6 times daily; F7: 7 times daily; F8: 8 times daily; F9: 9 times daily; Dim 1: dimension 1; Dim 2: dimension 2; X.0 to 10: fiber between 0 and 10 micrometers; X.10 to 20: fibers between 10 and 20; X.20 to 30: fibers between 20 and 30 micrometers; X.30 to 40: fibers between 30 and 40 micrometers.

According to the graph of the correspondence analysis (Figure 1), it can be seen that there was an association between fibers from 30 to 40 micrometers and F8 and F9. In F4, the fibers were the most distant in this category.

In the early stages of fish development, muscle fibers are predominantly multiplying [32], through the recruitment of satellite cells that go to the periphery for further maturation, while in hypertrophy, the nucleus of these cells is internalized by the growing fiber, causing fiber enlargement [33]. Although there is a prevalence of hyperplasia in the early stages and hypertrophy in the later stages, nutrition and other environmental factors can interfere with the dynamics of new fiber recruitment [33–35], which was observed in the present study.

The present data show that growth seems to have occurred in a mosaic pattern in all treatments, with smaller fibers surrounded by larger fibers, indicating that the animals will have continuous growth of muscle mass, and even F4, which was negatively correlated to larger fibers (Figure 1), may reach the size of the others when fed at the same proportion in future stages. However, it is immediately apparent that these fish were limited in the development of larger fibers (only 45 fibers between 30 and 40 *μ*m compared to 154, 140, 141, 204 and 230 of F5, F6, F7, F8, and F9, respectively), as their WG was also lower.

Fibers between 10 and 20 *μ*m prevailed in fish fed between four and seven times a day, decreasing at higher feeding frequencies (F8 and F9). Also, these two treatments had a higher occurrence of fibers between 30 and 40 micrometers, confirming the lower hyperplasia at high feeding frequencies. This parameter is of great relevance to producers that will work in growth phases with these fingerlings, because the fibers recruited in larger quantities (which occurred in F4, F5, F6, and F7) will be available for hypertrophy, which is responsible for muscle development, that is, the production of the fillets.

Regarding the liver, there was a significant difference between F9 and F5 (*p* = 0.0020) and F8 and F5 (*p* = 0.0234) in the analysis of the area of hepatocytes (Table 6), indicating that the frequency with which the fish are fed interferes with liver health, which is important for the survival and health of the produced batches. Previously, it was observed that the HSR was not changed, but this may have been due to the short time of the experiment. At the cellular level, this result indicates the beginning of liver alterations resulting from the higher feeding frequency.

Another observation is that in other studies, food restriction led to changes in the liver parenchyma, that is, long periods in which the fish seeks food and cannot be consumed [36]; this did not occur in this work, showing that it is possible to feed the fish only four times a day without harming their liver health; therefore, the lower frequency (F4), under this parameter, cannot be considered restrictive.

**Figure 2:**
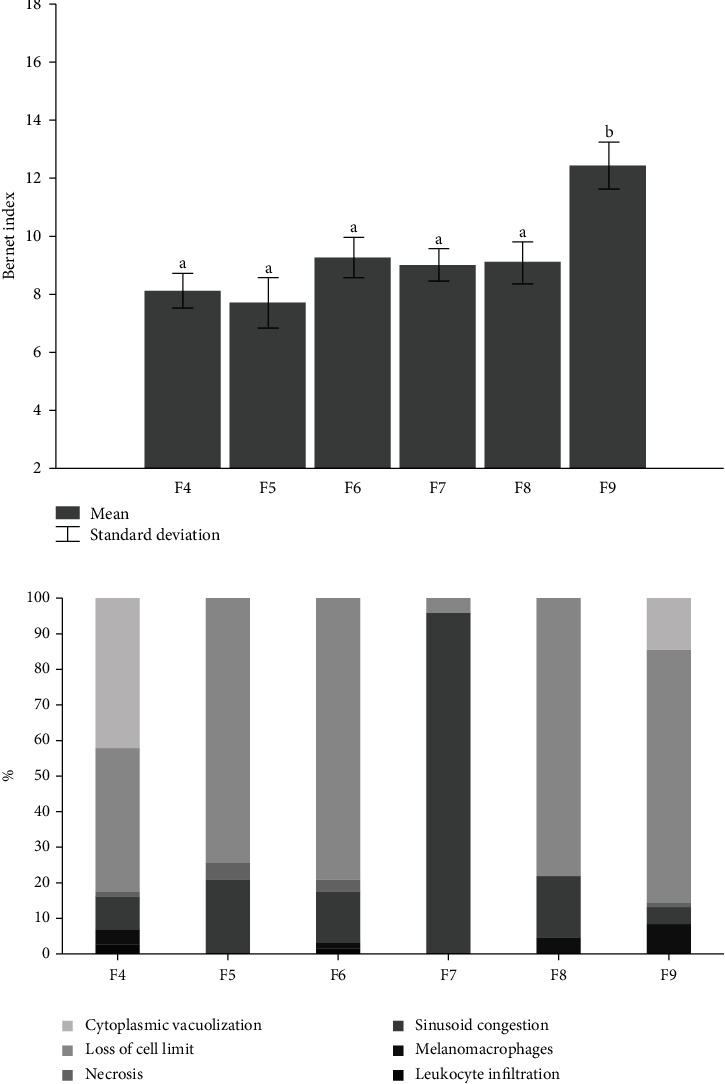
Bernet index (a) and index of histopathological lesions (b) of Nile tilapia fingerlings (*Oreochromis niloticus*) fed on F4 diets: fed 4 times a day; F5: fed 5 times a day; F6: fed 6 times a day; F7: fed 7 times a day; F8: fed 8 times a day; and F9: fed 9 times a day.

As for liver morphology, there was a difference for fish fed on the F9 diet, with more moderate lesions and more severe lesions in liver integrity (Figure 2).

As the liver is the organ responsible for processing the ingested food, which has its nutrients absorbed in the intestine and forwarded to the liver through the hepatic portal vein [31], the overfeeding provided in T9, led to injuries resulting from its overload. The other frequencies did not impact these parameters. This fact is important for the survival of the lots of fish and their health, because the fish often need to be transported in stressful situations that can aggravate the condition, and their performance in the growth phases can be impaired, since the liver is directly related to the body's response to food [31].

**Figure 3:**
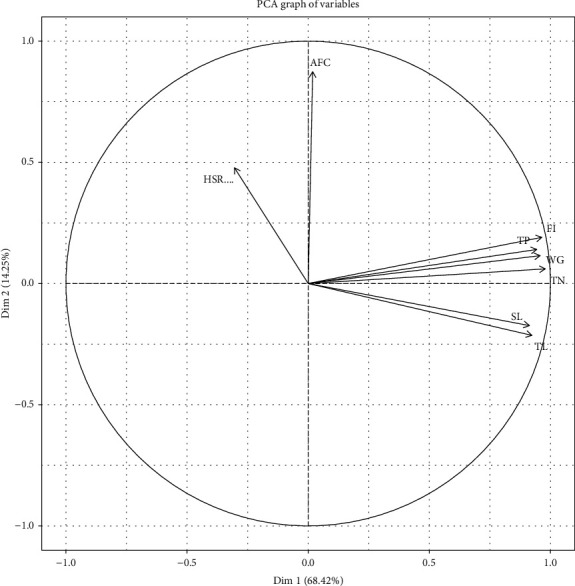
Principal component analysis (PCA) graph of variables. Graph with the vectors of the variables used in the study and main components 1 and 2, dim 1 and dim 2, respectively, the arrows indicate the load of the parameters in the two axes. HSR: hepatosomatic relation; AFC: apparent feed conversion; FI: feed intake; TP: total phosphorus; WG: weight gain; TN: total nitrogen; SL: standard length; TL: total length.

In PCA, the PC1 and PC2 axes explained 68.42 and 14.25% of the variation, totaling 82.67%. PC1 was positively associated with TP, FI, TN, WG, SL, and TL and negatively with HSR (Figure 3). The processes were classified with their biological (such as WG and AFC) and chemical (such as N and P) variables, arriving at the approach that assessed the interrelation of this large number of parameters and explaining them in two dimensions, which is the objective of the PCA according to Gallo et al. [37].

During the study, similarities were found between treatments by univariate analyses, but some trends were observed, which revealed the superiority of feeding between F5 and F6; however, in several parameters, this was not clear, and in most of them, there was no difference. By PCA, it was possible to reveal the different behavior of the fishes that were fed in F5 and F6, separating it from the others in Figure 3, and they were (F5 and F6) negatively associated from F4 in the same figure. F9 was between F5 and F6 and F7 and F8; all of them negatively associate with F4.

In the partial economic analysis (Table 7), it was observed that the highest PNR occurred in F6, the same treatment that, alongside F5, obtained the greatest weight gains. As the ration of all treatments presented the same value, the determining factors in the PNR were the expenses with ration (which is directly related to the AFC) and the gain obtained from the sale of the fry lots. The sample size did not allow the identification of statistical differences between the treatments at 5% but demonstrated at 10% (*p* = 0.0569), with a statistical difference occurring by comparing the means also at 10%, between F4 and F5 (*p* = 0.0812) and F4 and F6 (*p* = 0.0568).

In a study by Trombeta et al. [38], it was observed that fingerlings accounted for 4.97% of variable costs in a fish farm, while feed represented 63.78%, which shows the importance of this study. This is because the frequencies of F5 and F6 will result in greater gains in this 4.97% and, within this, in 63% of food costs, in other words, directly impacting the results of the complete production cycle.

The study presents results from the lower to high feed frequencies and its impacts on zootechnical performance, on liver and muscle histology, and on water quality parameters. Based on the results, F5 and F6 frequencies are the most suitable for the production of tilapia fingerlings among those tested. The impact on water quality was similar for treatments, but the results showed the best economic and zootechnical parameters to F4 and F5. Other than that, none of the treatments affected the health of the lots of fingerlings produced, although the resistance to other process must be investigated in future studies, as well as the performance of juveniles produced in these frequencies in other stages of development.

## 4. Conclusion

The frequency of feeding interfered in the metabolism of the fingerlings, making the fish fed between five and six times a day to obtain greater weight gain. Considering this factor and feed costs, these frequencies showed the best economic responses. It was also possible to observe liver changes due to high frequency feedings, with damage to fish fed 9 times a day. Through these results, it is possible to recommend the feeding of tilapia fingerlings five to six times a day.

## Figures and Tables

**Table 1:** Formulation and bromatological composition of feeds offered to Nile tilapia fingerlings (*Oreochromis niloticus*).

Ingredient	Inclusion (g kg^−1^)	Ingredient	Inclusion (g kg^−1^)
Corn bran	257.5	Calcitic limestone	5.6
Soil protein concentrate	223.9	L-Threonine	5.5
Rice bran	100.0	Salt	5.0
Poultry offal meal	100.0	Dl-methionine	3.8
Feather flour	68.0	Vitamin C	2.0
Corn gluten 60%	50.0	Choline chloride	1.5
Blood flour	50.0	Calcium propionate	1.0
Soy oil	33.3	Antioxidant (BHT)	0.2
Yeast alcohol	20.0	Total	1000.2
Dicalcium phosphate	17.7	Chemical composition (g kg^−1^)	
Fish meal 55%	16.7	DM	935.20
Hydrolyzed chicken protein	16.7	CP	390.30
Wheat gluten	10.0	EE	70.00
Mineral and vitaminic supplement	6.0	MM	84.40
L-Lysine HCL	5.8	EB	4.293

^∗^All ingredients were purchased at the local market. DM: dry matter; CP: crude protein; EE: ether extract; MM: mineral matter; GE: gross energy. Warranty levels per kilogram of product (mineral and vitaminic supplement): vit. A: 1.750.000 UI; vit. D3: 375000 UI; vit. K3: 500 mg, vit. B1: 2000 mg; vit. B2: 2500 mg; vit. B6: 2500 mg; vit. B12: 5000 mg; folic acid: 625 mg; calcium pantothenate: 7500 mg; vit. C: 37500 mg; biotin: 50 mg; inositol: 12500 mg; niacin: 8750 mg; choline: 100000 mg; cobalt: 50 mg; copper: 1250 mg; iron: 15000 mg; iodine: 100 mg; manganese: 3750 mg; selenium: 75 mg; zinc: 17500 mg.

**Table 2:** Water quality parameters presented as mean ± standard deviation. Temperature (T), dissolved oxygen (DO), electrical conductivity (EC), and hydrogen potential (pH) of the experimental units.

Variable	Feeding frequencies					
	F4	F5	F6	F7	F8	F9
T (°C)	25.0 ± 0.3	25.8 ± 0.2	25.5 ± 0.2	25.4 ± 0.4	25.6 ± 0.3	25.4 ± 0.3
DO (mg L^1^)	7.02 ± 0.11	7.20 ± 0.10	7.51 ± 0.07	7.15 ± 0.06	7.21 ± 0.22	7.17 ± 0.3
EC (*μ*S cm^−2^)	160.1 ± 0.07	169.7 ± 0.05	155.9 ± 0.08	160.1 ± 0.06	162.4 ± 0.03	161.1 ± 0.5
pH	7.62 ± 0.04	7.50 ± 0.02	7.49 ± 0.03	7.60 ± 0.05	7.55 ± 0.03	7.53 ± 0.4

^∗^Averages were not different according to Tukey's test (*p* > 0.05). F4: 4 times a day; F5: 5 times a day; F6: 6 times a day; F7: 7 times a day; F8: 8 times a day; F9: 9 times a day.

**Table 3:** Total nitrogen (TN) and total phosphorus (TP) in water during the experimental period with Nile tilapia (*Oreochromis niloticus*).

Variable (mg L^−1^)	Feeding frequency					
	F4	F5	F6	F7	F8	F9
T nitrogen	0.67 ± 0.06^b^	1.00 ± 0.21^a^	1.03 ± 0.15^a^	0.85 ± 0.07^ab^	0.86 ± 0.17^ab^	0.97 ± 0.16^ab^
T phosphorus	0.28 ± 0.03^b^	0.44 ± 0.08^ab^	0.46 ± 0.09^a^	0.34 ± 0.07^ab^	0.41 ± 0.07^ab^	0.45 ± 0.08^a^

^∗^Values without letters or with the same letters, which are on the same line, indicate that there was no significant difference (*p* > 0.05), whereas those with different letters indicate a significant difference (*p* < 0.05). F4: Fed 4 times a day; F5: fed 5 times a day; F6: fed 6 times a day; F7: fed 7 times a day; F8: fed 8 times a day; F9: fed 9 times a day.

**Table 4:** Mean values ± standard deviation of individual weight gain (EG), feed intake (FI), apparent feed conversion (AFC), final biomass (FB), total length (TL), standard length (SL), and hepatosomatic relation (HSR) of Nile tilapia fingerlings (*Oreochromis niloticus*) in different treatments.

Feeding frequency						
Variable^∗^	F4	F5	F6	F7	F8	F9
WG(g)	6.37 ± 0.4^b^	9.13 ± 1.9^a^	9.27 ± 1.72^a^	7.67 ± 0.72^ab^	7.80 ± 1.67^ab^	8.86 ± 1.40^ab^
FI (g)	80.70 ± 4.75	116.02 ± 24.69	115.93 ± 25.68	98.56 ± 9.66	100.38 ± 22.23	110.36 ± 17.98
AFC	1.29 ± 0.01	1.27 ± 0.03	1.24 ± 0.07	1.28 ± 0.03	1.28 ± 0.02	1.29 ± 0.01
TL (cm)	7.54 ± 0.36	8.64 ± 0.41	8.54 ± 0.36	8.13 ± 0.12	8.13 ± 0.50	8.42 ± 0.57
SL (cm)	5.94 ± 0.32	6.99 ± 0.38	6.92 ± 0.32	6.55 ± 0.15	6.61 ± 0.47	6.86 ± 0.47
HSR (%)	2.17 ± 0.16	2.11 ± 0.24	2.35 ± 0.37	2.13 ± 0.16	2.16 ± 0.32	2.36 ± 1.00

^∗^Values without letters or with the same letters, which are on the same line, indicate that there was no significant difference (*p* > 0.05), whereas those with different letters indicate a significant difference (*p* < 0.05). F4: Fed 4 times a day; F5: fed 5 times a day; F6: fed 6 times a day; F7: fed 7 times a day; F8: fed 8 times a day; F9: fed 9 times a day.

**Table 5:** Contingency table of frequencies and distributions of muscle fibers in three diameter classes (0-10 *μ*m, between 10 and 20 *μ*m, between 20 and 30 *μ*m, and between 30 and 40 *μ*m) in Nile tilapia fingerlings.

Feeding frequency						
Diameter classes	F4	F5	F6	F7	F8	F9
0-10 *μ*m	122	109	110	126	124	110
10-20 *μ*m	683	676	641	638	540	504
20-30 *μ*m	548	452	496	485	506	528
30-40 *μ*m	45	154	140	141	204	230

F4: fed 4 times a day; F5: fed 5 times a day; F6: fed 6 times a day; F7: fed 7 times a day; F8: fed 8 times a day; F9: fed 9 times a day.

**Table 6:** Mean values ± standard deviation of hepatocyte area (*μ*m^2^) and nuclear area (*μ*m^2^) in hepatocytes of Nile tilapia fingerlings (*Oreochromis niloticus*) in the different treatments.

Feeding frequency						
Morphometric parameters	F4	F5	F6	F7	F8	F9
Hepatocyte area (*μ*m^2^)	96.0 ± 13.1^ab^	114.1 ± 15.3^b^	98.2 ± 6.3^ab^	96.4 ± 18.5^ab^	82.1 ± 20.0^a^	72.9 ± 15.4^a^
Nuclear area (*μ*m^2^)	13.5 ± 0.8	14.00 ± 1.9	13.3 ± 2.00	12.9 ± 1.3	12.9 ± 1.3	12.8 ± 0.9

^∗^Values without letters or with the same letters, which are on the same line, indicate that there was no significant difference (*p* > 0.05), whereas those with different letters indicate a significant difference (*p* < 0.05). F4: Fed 4 times a day; F5: fed 5 times a day; F6: fed 6 times a day; F7: fed 7 times a day; F8: fed 8 times a day; F9: fed 9 times a day.

**Table 7:** Gross revenue (GR), costs (c), and partial net revenue (PNR) in the feed frequencies.

Feeding frequency						
Variable	F4	F5	F6	F7	F8	F9
GR (USD)	6.37	8.83	8.96	7.53	7.64	8.59
C (USD)	1.15	1.64	1.64	1.40	1.42	1.56
PNR (USD)	5.16	7.22	7.35	6.15	6.25	6.78

F4: four times a day; F5: five times a day; F6: six times a day; F7: seven times a day; F8: eight times a day; F9: nine times a day; USD: United States Dollar.

## Data Availability

Data used in the study will be available.
